# Social Stress Increases Anxiety-Like Behavior Equally in Male and Female Zebrafish

**DOI:** 10.3389/fnbeh.2021.785656

**Published:** 2021-12-20

**Authors:** Brenno Bozi, Jeane Rodrigues, Monica Lima-Maximino, Diógenes Henrique de Siqueira-Silva, Marta Candeias Soares, Caio Maximino

**Affiliations:** ^1^Laboratório de Neurociências e Comportamento “Frederico Guilherme Graeff”, Faculdade de Psicologia, Instituto de Estudos em Saúde e Biológicas, Universidade Federal do Sul e Sudeste do Pará, Marabá, Brazil; ^2^Grupo de Pesquisas em Neurociências, Comportamento & Cognição, Universidade Federal do Sul e Sudeste do Pará, Marabá, Brazil; ^3^Programa de Pós-Graduação em Reprodução Animal da Amazônia, ReproAmazon—Universidade Federal do Pará, Belém, Brazil; ^4^Grupo de Estudos da Reprodução Animal, Universidade Federal do Sul e Sudeste do Pará, Marabá, Brazil; ^5^Departamento de Morfologia e Ciências Fisiológicas, Universidade do Estado do Pará, Campus VIII, Marabá, Brazil; ^6^CIBIO/InBIO—Centro de Investigação em Biodiversidade e Recursos Genéticos, Universidade do Porto, Vairão, Portugal

**Keywords:** psychosocial stress, dominance-subordinate behavior, social plasticity, zebrafish, anxiety-like behavior

## Abstract

Zebrafish anxiety-like behavior was assessed in the novel tank test after the formation of dominant-subordinate hierarchies. Ten pairs of animals were subjected to dyadic interactions for 5 days, and compared with control animals. After this period, a clear dominance hierarchy was established across all dyads, irrespective of sex. Social status affected parameters of anxiety-like behavior in the novel tank test, with subordinate males and females displaying more bottom-dwelling, absolute turn angle, and freezing than dominant animals and controls. The results suggest that subordinate male and female zebrafish show higher anxiety-like behavior, which together with previous literature suggests that subordination stress is conserved across vertebrates.

## Introduction

Whenever established between animals, dominance hierarchies usually produce relevant behavioral adaptations in both dominant and subordinate individuals. These adaptations are examples of social plasticity, which is the capacity to adaptively change behavior according to previous experience and to social context (Maruska et al., 2019). Across fish species, dominance hierarchies have been shown to increase the fitness of dominant individuals by allowing greater access to resources and mating opportunities (Oliveira, 2012; Maruska et al., 2019). However, little is known about the effects of dominant-subordinate interactions on the behavior of subordinate individuals.

In rodents, the experience of subordination is sometimes used as a model of psychosocial stress, as many stressors that are relevant to human mental health involve experiencing social subordination (Blanchard et al., 1993; Read and Harper, 2020). Another relevant model organism for behavioral neuroscience and biological psychiatry is the zebrafish (Stewart et al., 2014), which has been widely used in behavioral tests and as a model of anxiety-like behavior (Kysil et al., 2017). In this regard, zebrafish seem to be an ideal fish species to understand the effects of social subordination on anxiety-like behavior.

Moreover, neurobehavioral and physiological changes have been observed to occur in subordinate zebrafish, regarding pathways associated with stress responses. For instance, male subordinate zebrafish showed higher plasma cortisol levels, as well as higher expression of corticotropin-releasing hormone, neuropeptide Y, and glucocorticoid receptor in the telencephalon (Filby et al., 2010a). On the other hand, subordinate male zebrafish exhibited elevated plasma cortisol concentrations and impaired neurogenesis in the dorsal but not ventral regions of the telencephalon, an effect that was attenuated by treatment with metyrapone, a cortisol synthesis inhibitor (Tea et al., 2019). Both male and female subordinate zebrafish showed higher serotonergic activity in the hindbrain (Dahlbom et al., 2012). Finally, subordinate males showed an up-regulation in the expression of glucocorticoid and mineralocorticoid receptors, brain-derived neurotrophic factor (BDNF), the 5-HT2B receptor, and isoform B of the serotonin transporter (Theodoridi et al., 2017). Overall, these findings suggest that the experience of subordination activates pathways related to arousal and defensive (anxiety-like) behavior in zebrafish, which is likely to lead to increased anxiety-like behavior.

But are sex differences significant to both the establishment of dominance hierarchies and to the effects of this particular psychosocial stressor? From the point of view of psychopathology, human responses to threat more closely resemble the effects of social subordination (e.g., anxiety and depression) and are more prevalent in women (Riecher-Rössler, 2017), with women also more likely to suffer social violence and imposed subordination than men in Western societies (Oram et al., 2017). From the point of view of animal behavior, this question remains relevant because, in spite of anthropocentric biases, female individuals have been shown to enter dominance hierarchies in many species, including zebrafish (Filby et al., 2010a; Paull et al., 2010; Dahlbom et al., 2012; Tea et al., 2019). In zebrafish, anxiety-like behavior has been described in a now growing amount of tests, the most used of which are the light/dark test (Maximino et al., 2010) and the novel tank test (Levin et al., 2007; Bencan et al., 2009; Egan et al., 2009). In the latter, a neophobic response is elicited by introducing the fish to a novel tank and usually is observed as bottom-dwelling (i.e., spending most of the trial in the bottom of the tank) associated with freezing and erratic swimming (Egan et al., 2009). The main controlling stimulus in the cast of the novel tank test is escape from the top of the tank (Maximino et al., 2012), which could be related to antipredator strategies employed by zebrafish in the wild (Engeszer et al., 2007). Moreover, the novel tank test is relatively sensitive to anxiolytic and anxiogenic manipulations (Kysil et al., 2017), showing potential as a way of detecting the effects of social stress on anxiety-like behavior, as well as sex differences in these effects.

While female zebrafish have been observed to form dominant-subordinate hierarchies with other females, neurochemical effects appear to be different in female subordinates compared to males; for example, Tea et al. (2019) observed the formation of hierarchies among female zebrafish without elevations in plasma cortisol and changes in neurogenesis, differently from males. Likewise, Filby et al. (2010a) did not observe, in female subordinate zebrafish, any changes in the expression arousal of pathway genes that were also observed in males. Thus, sex differences in the physiological consequences of psychosocial stress suggest that female zebrafish would be less sensitive than males to possible anxiogenic-like effects of subordination stress.

## Methods

### Animals and Housing

Ten pairs of adult zebrafish (standard length: 36.1 ± 0.1 mm) were used in the present experiment for tournaments. Fifteen other animals were used as controls (i.e were not exposed to dyadic encounters, being transferred to the experiment room directly from the home tank). Animals were obtained from a commercial breeder and maintained in the laboratory for at least 4 weeks before experiments began. Animals were from a wild-type population with the longfin phenotype; outbred populations were used for increased genetic variability, decreasing possible effects of random genetic drift that could lead to the emergence of uniquely heritable traits that could affect behavior (Parra et al., 2009). The breeder was licensed for aquaculture under Ibama’s (Instituto Brasileiro do Meio Ambiente e dos Recursos Naturais Renováveis) Resolution 95/1993. While the breeding status of animals brought to the laboratory cannot be ascertained, breeding pairs were never established in the laboratory before experiments began. Animals were kept in mixed-sex tanks during acclimation in 40 L tanks, with a maximum density of 25 fish per tank. Fish were held under a 14L:10D photoperiod, and water parameters were held at recommended levels for welfare conditions (Lawrence, 2007), in non-chlorinated water at room temperature (28°C) and a pH of 7.0–8.0. Water quality parameters were as follows: pH 7.0–8.0; hardness 100–150 mg/L CaCO_3_; dissolved oxygen 7.5–8.0 mg/L; ammonia and nitrite <0.001 ppm. All manipulations were carried out in a bid to minimize the potential suffering of fish, and followed Brazilian legislation (Diretriz brasileira para o cuidado e a utilização de animais para fins científicos e didáticos—DBCA. Anexo I. Peixes mantidos em instalações de instituições de ensino ou pesquisa científica, Resolução Normativa CONCEA n^o^ 34 218, 2017). Animals were solely used in one experiment and one behavioral test, to reduce interference from apparatus exposure. Experiments were approved by UEPA’s IACUC under protocol 06/18.

### Establishing a Hierarchy Through Dyadic Encounters

Dyadic encounters were established following a protocol adapted from (Filby et al., 2010a, b) . Briefly, immediately prior to artificial dusk on day 0, 20 fish were randomly assigned into 10 experimental tanks (20 cm × 14.5 cm × 12.5 cm), with two animals per tank, and allowed to acclimatize overnight so that fish could recover from any handling stress and then resume normal behavior, and therefore being able to develop a dominance hierarchy. Animals were kept in the experimental tanks for the subsequent 5 days, a period that is sufficient to establish dominance hierarchies in most zebrafish (Filby et al., 2010a,b; Paull et al., 2010). An *a priori* stopping rule was established in that, if animals displayed signs of poor health in any of the 5 days of hierarchy establishment, the experiment would end for that pair, and the animals substituted for another pair, with the initial individuals treated for any putative wounds or health-derived parameters. However, none of the pairs were swapped during this study. On the morning of the 5th day, the experimental tanks were videotaped simultaneously for 5 min., and recordings were later transcribed, using X-Plo-Rat^1^ to assess the incidence of dominant and/or subordinate behaviors. Following Pavlidis et al. (2011), the following endpoints were assessed:

• Chasing (N): The number of chasing events throughout the 5 min session, defined as the fish “swim[ming] directly/aggressively toward another fish in the aquarium causing it to increase its speed (and possibly change direction) and actively pursue[ing] the fish” (Paull et al., 2010, p. 111);
• Bottom-dwelling (s): The time spent in the bottom third of the tank;
• Immobility (s): The total duration spent in “a motionless state during which only the gills and, occasionally the eyes may move” (Blaser and Gerlai, 2006, p. 457); and
• Attacks (N): The number of events in which one fish attacked the other, with or without being associated with biting. It iIncludes both spars and bites (Paull et al., 2010).

Immediately after the end of the recording, animals were observed individually in the novel tank test (NTT, see section below) to assess anxiety-like behavior. Control animals were not exposed to dyadic encounters, instead they were transferred directly from the home tank to the experiment room. The order of individuals tested in NTT trials was randomized *via* generation of random numbers using the randomization tool in http://www.randomization.com/. Experimenters at this stage were blinded to social status.

**Figure 1:**
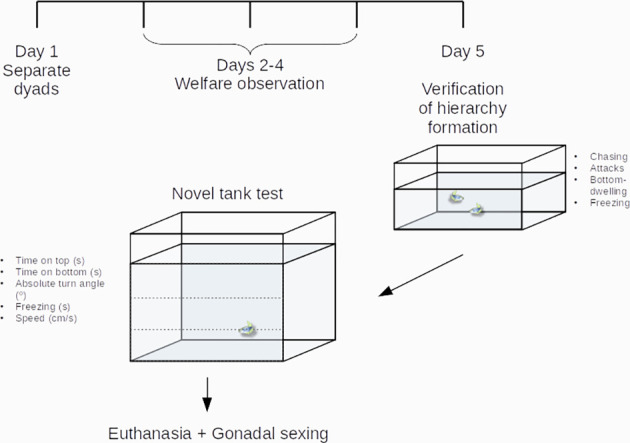
Experimental design. Zebrafish dyads were established on Day 1, had welfare endpoints checked during Days 2–4, after which hierarchy formation was verified on Day 5. After behavioral observations of aggressive and territorial behavior, animals were transferred and observed individually in the novel tank test. See the “Methods” section for more information.

After video transcription, dominant status was ascribed to the fish in a single pair that showed the highest frequency of chasing and attacks and the lowest time spent freezing and bottom-dwelling, while subordinate status was ascribed to the fish that showed the lowest frequency of chasing and attacks and the highest time spent freezing and bottom-dwelling (Figure 1).

### Novel Tank Test

Anxiety-like behavior was assessed after the last encounter in the novel tank test (Levin et al., 2007; Bencan et al., 2009; Egan et al., 2009), an assay for anxiolytic-like and anxiogenic-like effects that is relatively sensitive to pharmacological (Kysil et al., 2017) and contextual (Tran and Gerlai, 2016) manipulations. The novel tank test followed a protocol used in our laboratory. Briefly, after the last encounter, animals were individually transferred to a transparent glass aquarium (5 cm × 24 cm × 20 cm) filled with 5 l of mineral water where the animal could freely explore the space for a period of 6 min during which their behavior was recorded, using a video camera (Sony^®^ DCR-DVD610). The camera was positioned in the front of the tank, therefore allowing observation and tracking of vertical distribution. All stages of the experiment were performed under constant white Gaussian noise, producing an average of 58 dB above the tank. Light levels above the tanks were measured using a handheld light meter and ranged from 249 to 281 lumens (coefficient of variation = 4.1% between subjects). Video files for each experiment were stored and later analyzed using automated video tracking (TheRealFishTracker^2^). The following variables were extracted:

• Time spent on the bottom third of the tank (s)[Primary outcome]
• Time spent on the top third of the tank (s)[Secondary outcome]
• Erratic swimming, measured as absolute turn angle [Secondary outcome]
• Freezing (s), measured as time spent in a speed lower than 0.5 cm/s [Secondary outcome]
• Swimming speed (cm/s) [Secondary outcome]

### Gonadal Sexing

While external sexing in zebrafish is possible based on body shape, gonadal sexing was established to confirm external body-shape-based analyses. After the end of the NTT, animals were sacrificed by immersion in ice-cold water and fast spinal transection (Matthews and Varga, 2012) and fixed in Bouin’s fixative for 24 h. To facilitate infiltration of the fixative, a ventral incision was made into the body of the animal. Fixed tissues were included in meth-acrylate glycol (Historesin, Technovich, 7100). Serial sections were cut at 5 μm slices, collected onto glass slides, and stained with hematoxylin-eosin (Cordeiro et al., 2020). Analysis of slide preparations was performed with a Leica CTR4000 light microscope. The oocytes of the zebrafish were classified according to Selman et al. (1993). After sexing, it was determined that six dyads were of different sex individuals (two with a dominant female over subordinate male, four with dominant males over subordinate females), nine dyads were composed of two male individuals, and five dyads were composed of two female individuals.

### Statistical Analysis

To test the null hypothesis of independence between status and sex for same-sex and different-sex pairs, a permutation-based independence test using Cochran-Mantel-Hanszel quadratic (c_quad_) statistics (Hothorn et al., 2006), using the R package “coin” (v. 1.4–1; Hothorn et al., 2006,^3^) was applied. To test the null hypothesis that sex did not affect aggressive behavior on the last day of dyadic encounters, a multivariate, permutation-based partially ordered set (POSET) test (Rosenbaum, 1994) was applied to a coherence function containing all observed variables. The POSET test is based on a coherence criterion defining a partial ordering, i.e., an observation is smaller than another when all responses are smaller, and a score reflecting the “ranking” is attached to each observation. To test the null hypothesis that anxiety-like behavior was not affected by sex and status, a POSET test was applied to a coherence function of time on top, time on bottom, absolute turn angle, and freezing. POSET tests were made using the R package “coin”. Data were presented as Gardner-Altman estimation plots, with individual data points presented as a swarm plot and the effect size (mean difference) presented as a bias-corrected and accelerated bootstrap 95% confidence interval. Estimation plots were created using the R package DABESTR (Ho et al., 2019,^4^). All analyses and plots were made using R version 3.6.3. Data and analysis scripts can be found on a GitHub repository^5^.

## Results

After 5 days of co-housing, a clear dominance hierarchy was established across all dyads, irrespective of sex (*c*_quad_ = 0.599, *p* = 0.74, df = 2). Sex differences were found in the number of attacks, with females, in general, attacking more than males, but no differences were seen between males and females in other variables used to classify subjects as dominant or subordinate (i.e., chasing, bottom-dwelling, immobility, and retreats). A POSET-test for all variables did not find significant differences between males and females (*Z* = 0.6782, *p* = 0.619), reinforcing the idea that both male and female zebrafish can enter into dominant-subordinate hierarchies. Table 1 shows the effect sizes for each endpoint.

**Table 1:** Endpoints and effect sizes (unpaired mean differences between female and male individuals) in the observation session made on the 5th day of hierarchy formation across zebrafish.

Endpoint	Effect size (unpaired mean difference between females and males)
Chasing	−26.2, 95%CI [−52.5; −1.21]
Bottom-dwelling	8.11, 95%CI [−134; 155]
Attacks	51.7, 95%CI [20.7; 101]
Immobility	−17.6, 95%CI [8–83.6; 50]
Retreats	−20.2, 95%CI [−57.8; 20.7]

*Effect sizes are accompanied by 95% confidence intervals (CI), calculated by taking 5,000 bootstrap samples, bias-correcting, and accelerating (cf. Ho et al., 2019)*.

**Figure 2:**
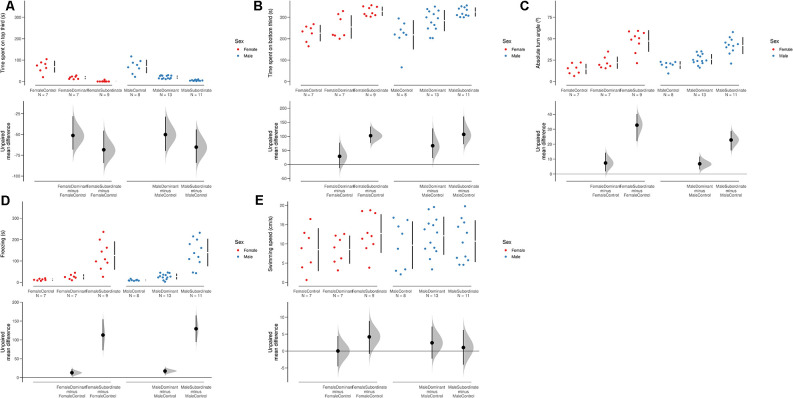
Swarmplots and Cumming estimation plots for variables in the novel tank test. **(A)** Time spent at the bottom third of the tank. **(B)** Time spent at the top third of the tank. **(C)** Absolute turn angle. **(D)** Freezing duration. **(E)** Swimming speed. The mean difference for three comparisons against the shared control (male controls) in the above Cumming estimation plots. The raw data is plotted on the upper axes. On the lower axes, mean differences are plotted as bootstrap sampling distributions. Each mean difference is depicted as a dot. Each 95% confidence interval is indicated by the ends of the vertical error bars. 5,000 bootstrap samples were taken; the confidence interval is bias-corrected and accelerated.

Stratification of status (dominant/subordinate) by sex revealed a significant effect on behavior in the NTT (POSET test, maxT = 6.175, *p* < 0.0001), with no effects of sex (step-down adjusted *p* = 0.649), but a significant effect of status (step-down adjusted *p* < 0.0001). Overall, subordinates spent more time at the bottom of the tank (Figure 2A), less time at the top of the tank (Figure 2B), increased absolute turn angles (Figure 2C), and increased freezing (Figure 2D) than both controls and dominant individuals, but no differences in swimming speed were observed (Figure 2E).

## Discussion

The present work tested the hypothesis that status in a dominance-subordinate hierarchy increases anxiety-like behavior in the zebrafish. Overall, both male and female zebrafish were capable of establishing dominance, with subordinate males and females displaying increased anxiety-like behavior in the novel tank test after the establishment of the hierarchy.

While adaptive changes in the physiology of subordinate zebrafish has been observed before, which are consistent with increased defensive/anxiety-like behavior, especially in the up-regulation of expression and activity of the hypothalamus-pituitary-internal (stress) and serotonergic pathways, behavioral adjustments have not been reported before. Dahlbom et al. (2011) reported that zebrafish with a “bolder” profile, spending more time in the center of an open tank and being more active in the novel object test, were more likely to become dominant in following tournaments, suggesting that pre-existing differences in anxiety-like behavior in regards to novelty are predictive of the animal’s ability to attain dominance. Indeed, a boldness-aggression syndrome has been repeatedly observed in zebrafish across stocks, strains, and contexts (Moretz et al., 2007; Norton et al., 2011; Ariyomo et al., 2013; Martins and Bhat, 2014; Mustafa et al., 2019), suggesting that lower anxiety-like behavior is associated with higher aggression, which in its turn is predictive of the capacity to become dominant. However, in the present study subordination elicited increased anxiety-like behavior in the NTT in comparison to controls, suggesting that heightened anxiety was not a pre-existing difference across conditions.

Interestingly, both males and females were capable of establishing hierarchies. While the number of mixed-sex pairs was relatively small, and therefore it’s not possible to ascertain whether male-female dyads consistently produce hierarchies instead of simply leading to attempts to courtship and/or male coercion, in two of the pairs, the female was indeed dominant, patrolling the top of the tank and chasing the subordinate male. Further experiments are needed to confirm whether females commonly become dominant over males.

Differences between sexes were also apparently absent in the effects of subordination on anxiety-like behavior. Sex differences have been observed before in zebrafish in both directions. For example, while male subordinate zebrafish were observed to display higher cortisol levels and impaired neurogenesis, female subordinate zebrafish did not display such an effect (Tea et al., 2019), suggesting that females might not be susceptible to the anxiogenic-like effect of subordination stress. However, baseline anxiety-like behavior was observed to be higher in females than in male zebrafish (Genario et al., 2020). Both results, which point to different directions, are inconsistent with the apparent lack of effect of sex on anxiety-like behavior. Regarding effect sizes, it appears that female subordinates showed a larger difference in absolute turn angle (erratic swimming) than subordinate males, suggesting sex differences in specific behaviors. Direct and conceptual replication attempts at other laboratories and contexts could further clarify the robustness of the lack of sex differences in the anxiogenic-like effects of subordination in zebrafish.

Overall, the present study shows that, in zebrafish, subordination is a psychosocial stressor that can lead to increased anxiety-like behavior. This has been observed previously in mammals (Blanchard et al., 1993), an effect that has been further exploited as a model to understand the neurobehavioral effects of social stress and its relations to anxiety and depression. While previously subordinate zebrafish have been shown to display behaviors which are consistent with an anxiety-like state during dyadic encounters (Filby et al., 2010a), systematic changes in behavior in a different context (to rule out behavioral displays of submission) and in tests explicitly designed to assess anxiety-like behavior were not previously used. The convergent evidence from rodents and zebrafish referring to dominant/subordinate stress effects to anxiety-like subordinate behavior points towards the existence of a conserved trend across social species, which should be further exploited to understand the extent of similarity/dissimilarity of the underlying mechanisms (Gerlai, 2020).

## Data Availability Statement

The datasets presented in this study can be found in online repositories. The names of the repository/repositories and accession number(s) can be found below: https://github.com/lanec-unifesspa/zebrafish_subordination.

## Ethics Statement

The animal study was reviewed and approved by CEUA/UEPA.

## Author Contributions

BB: conceptualization, methodology, investigation, data curation, formal analysis, visualization, and writing—original draft. JR: conceptualization, investigation, and formal analysis. ML-M, DS-S, and MS: conceptualization, formal analysis, and writing—original draft. CM: conceptualization, formal analysis, funding acquisition, methodology, project administration, resources, software, supervision, validation, visualization, writing—original draft, writing—review and editing. All authors contributed to the article and approved the submitted version.

## Conflict of Interest

The authors declare that the research was conducted in the absence of any commercial or financial relationships that could be construed as a potential conflict of interest.

## Publisher’s Note

All claims expressed in this article are solely those of the authors and do not necessarily represent those of their affiliated organizations, or those of the publisher, the editors and the reviewers. Any product that may be evaluated in this article, or claim that may be made by its manufacturer, is not guaranteed or endorsed by the publisher.

## Footnotes

^1^ https://github.com/lanec-unifesspa/x-plo-rat

^2^ http://www.dgp.toronto.edu/~mccrae/projects/FishTracker/

^3^ https://cran.r-project.org/package=coin

^4^ https://CRAN.R-project.org/package=dabestr

^5^ https://github.com/lanec-unifesspa/zebrafish_subordination
